# Anticancer Activity of Ethnopharmacological Plants of Golestan Province/Iran against AGS, HT-29 and KYSE-30 Cell Lines through Promoting the Apoptosis and Immunomodulatory Effects

**DOI:** 10.22037/ijpr.2021.114451.14858

**Published:** 2021

**Authors:** Mahdieh Naghavi Alhosseini, Masoumeh Mazandarani, Ayesheh Enayati, Mohsen Saiedi, Homa Davoodi

**Affiliations:** a *Laboratory Sciences Research Center, Golestan University of Medical Sciences, Gorgan, Iran. *; b *Department of Botany, Islamic Azad University, Gorgan Branch, Gorgan, Iran. *; c *Ischemic Disorders Research Center, Golestan University of Medical Sciences, Gorgan, Iran. *; d *Stem Cell Research Center, Golestan University of Medical Sciences, Gorgan, Iran. *; e *Cancer Research Center, Golestan University of Medical Sciences, Gorgan, Iran.*; 1 * M. N. A. and A. E. contributed equally to this work.*

**Keywords:** Ethnopharmacological plants, Cytotoxicity, Gastrointestinal cancer, TLR-4, AKT/ ERK, NFκB

## Abstract

The anticancer and immunomodulatory effects of medicinal plants from Golestan province, as a promising source of cancer therapy against gastrointestinal cancer cell lines, were investigated in this study. The ethanolic root/aerial part extracts of 9 medicinal plants were screened for their cytotoxicity against normal mouse fibroblast cells (L-929) and three human cancer cell lines including gastric adenocarcinoma (AGS), colorectal adenocarcinoma (HT-29), and esophagus adenocarcinoma (KYSE-30) by performing MTT assay to determine the IC50 of the extracts. The *in-vitro *antioxidant activity, total phenolic (TPC), and total flavonoid content (TFC) of extracts was evaluated. Flow cytometry and Real-Time PCR were used for apoptosis assay and evaluation of expression of some genes involved in cell signaling*; TLR-4, AKT, ERK1/2*, and *NFκB*. Out of the 9 plant extracts screened, Arctiumlappa root (*ALR*), showed the most potent cytotoxicity against *AGS*, *KYSE-30*, and *HT-29* cells with IC50 values of 10, 200, and 2030 µg/mL, respectively. In addition, *ALR* exerts high TPC (215.8 ± 0.3 mg GAE/g), TFC (69.03 ± 0.7 mg QUE/g) and high radical scavenging activity with IC50 (1250 ‎±‎ 0.1 µg/mL) in DPPH method. Also, ALR stimulates TLR-4 signaling, increased apoptosis, and decreased cancer cell attachment to the surface compared to the untreated cells. This plant, with a strong cytotoxic effect on cancer cells as well as increased apoptosis and its effect on molecules involved in TLR4 signaling as the immunomodulatory effect can be a suitable candidate for in-vivo studies in the future for cancer therapy.

## Introduction

Despite the advanced techniques for the detecting and treatment of cancer, referring to the World Health Organization (WHO), cancer is the second factor of death globally, and is responsible for about 1 in 6 deaths in 2018. Among different cancers, gastrointestinal cancers such as esophageal, stomach, and colorectal are the most common cancers in the world (1, 2). At present, there are various methods using for cancer treatment such as chemotherapy but they have limited effects because of the no-selectivity of medicines and resistance of tumor cells to the drugs (3-6). The most important point in cancer treatment is eradicating tumor cells, without damaging normal cells. To overcome these limitations, it is essential to find potent new cytotoxic and anticancer drugs to control cancers. 

Nowadays, medicinal plants as a promising source of cancer therapy could cause multiple beneficial effects on tumor cells along with little or no toxicities to patients (7, 8). Herbal medicines by their active ingredients such as polyphenols (9), flavonoids (10), triterpenoids, acetylene, and sulforaphane, could ameliorate the damage of DNA and stimulate antitumor enzymes, anti-inflammatory effects, enhance immunity, modulate cell adhesion, and decrease cell proliferation and antioxidant effects (11-14). Natural phytochemicals such as taxol, paclitaxel, vinblastine, and vincristine were the first-line therapeutic agents in conventional medicine for cancer treatment (8). Some studies indicated that medicinal plants and their isolated ingredients trigger the expression of some specific genes as well as signaling cascades in cancer treatment (15-17). 

Toll-like receptors (*TLRs*) are known as the receptors of damage-associated molecular patterns and pathogen-associated molecular patterns (18). Among these receptors, TLR4 is the most extensively studied TLR which is known to mediate the innate immune response in inflammatory disorders such as cancers. (19). Mitogen-activated protein kinase (ERK1/2) and AKT protein kinases the major downstream pathways involved in inflammation and autoimmune diseases mediate the apoptotic effect of natural products in cancer cells. Many previous studies have demonstrated the regulation of TLR-4, AKT and ERK1/2 expression in cancer cell lines in response to herbal extracts treatments (16, 17 and 20). Previous studies reported that lycopene (a carotenoid) showed its anti-cancer effect on HT-29 cell lines via inhibiting Akt phosphorylation. Along with this, triterpenoids decrease the p-Akt and p- ERK1 levels for inducing apoptosis on cancer cells (21, 22).

Golestan province is rich in medicinal plants. In this study, several plants with a history of medicinal use were studied for anti-tumor properties. Although there are reports of their anti-tumor properties in other geographical regions, this study was conducted because the same plant species under different ecological conditions produce different primary and secondary metabolites. Since gastrointestinal cancers are among the most common cancers in the Golestan province, this study was performed to determine the antitumor effects of total alcoholic extract of these plants on gastrointestinal cancer cells. 

The cytotoxic and antioxidant activities, cell adhesion, apoptotic effects, and the expression of the genes involved in cell signaling; TLR-4, AKT, ERK1/2 and NFκB were investigated in colorectal, esophageal and stomach cancer cell lines.

## Experimental

*Plant materials*

The aerial parts and roots of 9 plants were collected in June-July in 2018 from Golestan province, Iran (Table 1). The selection of plants was based on different literature sources, folklore and traditional medicine. The plants were taxonomically identified by Professor Masumeh Mazandarani at the Central Herbarium of Islamic Azad University, Gorgan, Iran and voucher specimens were kept in the herbarium.

*Preparation of plant extracts*

The aerial parts and roots were dried at room temperature and then powdered in a mechanical grinder. The powder samples were macerated with 80% ethanol for 3 days by shaking at room temperature (23). The extracts were filtered and then concentrated using a rotary evaporator under 45 ºC and stored at -80 ºC for further use and testing.

*Total Phenolic Content*

Folin-Ciocalteu colorimetric method was used to measure the total phenolic content (TPC) of *Arctium lappa *extracts, as previously described (24). In summary, a Folin-Ciocalteu reagent (Merck, Germany) was added to the methanol solution of *A. lappa* aerial part/root extracts. Then, Na_2_CO_3 _solution (1.5 mL, 60 g/L distilled water) was added and the absorption of the solutions was measured at 725 nm after 90 min at 22 °C. Gallic acid was used as a standard calibration curve. TPC was expressed as milligrams of gallic acid equivalents (GAE) per gram of dried extracts. 

*Total Flavonoid Content*

The content of flavonoids (TFC) was determined by the aluminum chloride colorimetric method and quercetin was used as a standard. Based on the protocol, 0.5 mL of extract or standard was diluted by 1.5 mL of solvent and then 0.1 mL of 10% aluminum chloride, 0.1 mL of 1 mol/L potassium acetate, and 2.8 mL of distilled water were added to the diluted samples. After 30 min, absorbance was measured at 415 nm with a spectrophotometer. Total flavonoid contents were reported in terms of mg equal quercetin in a one-gram powder dry plant (25).

*DPPH free radical-scavenging activity assay*

Free radical-scavenging potentials of the aerial part and root extracts of *Arctium lappa *were determined spectrophotometrically by the DPPH assay, as previously described (24). Briefly, different concentrations of the extract were prepared, then 1ml of each diluted extract solution was added to 2 mL of DPPH solution. Subsequently, absorptions were measured at 517 nm after remaining at 25 °C in a dark environment for 30 min. IC_50_ values were reported as Mean ± SD.

*Cell Culture*

Four different cell lines, *L929* (mouse fibroblast-like cell line, *NCBI#: C161*), *AGS* (human gastric adenocarcinoma cell line, NCBI#: C131), *HT-29* (human colon adenocarcinoma cell line, NCBI#: C466 and *KYSE-30* (human esophageal squamous cell carcinoma cell line, *NCBI*#: C584) were applied for cytotoxicity screening of the Golestan province local plant extracts. All cell lines were from Pasteur Institute, Tehran, Iran. Cell lines were cultured in liquid medium *RPMI1640* and *DMEM* (*Gibco, Carlsbad, USA*) supplemented with 10% Fetal Bovine Serum (Gibco, Carlsbad, USA), 100 u/ml penicillin (Gibco, Carlsbad, USA) and100 μg/ml streptomycin (Gibco, Carlsbad, USA) and grown at 37 ºC in a humidified atmosphere of 5% CO_2_ in the air.

*Cytotoxic Activity (MTT Assay* ***)***

Phosphate buffer saline (*Sigma, St. Louis, USA*) washed cells harvested by trypsinization (*Gibco, Carlsbad, USA*) were then plated in 96-well plates with 1 × 10^4^cells/well of concentration and incubated under 5% CO_2_ and 95% air at 37 ºC for 24 h. The cells were treated with different concentrations of plants extracts including 31.2, 62.5, 125, 250, 500,1250, 2500, 5000,10000, 20000, and 40000, 80000, 160000 µg/mL. Dilution of stock solutions was made in culture medium yielding final extracts concentrations with a final DMSO (Sigma, St. Louis, USA) concentration of less than 0.1%. All concentrations were done in duplicate.

 The cytotoxicity of herbal extracts was quantitated by the ability of live cells to reduce the yellow dye 3- (4, 5-dimethyl-2-thiazolyl)-2, 5-diphenyl-2H-terazolium bromide (MTT) to a blue formazan product (26). Incubation time was assessed in 24, 48 and 72 h but the optimum time to calculate IC_50_ was 48 h.

After 48h incubation time, the medium in each well was replaced by MTT solution (5 mg/mL in phosphate-buffered saline), the plates were incubated for 4 h under 5% CO_2_ and 95% air at 37 ℃. MTT reagent was removed and the formazan crystals produced by viable cells were dissolved in *DMSO*. The absorbance was then determined by the *ELISA* reader at 570 nm. The percentage growth inhibition was calculated using the following formula:

Viability of cells = Sample OD/Control OD × 100

The effects of extracts were expressed by *IC*_50_ values (the *IC*_50_ value was defined as the concentration of compound where percentage inhibition is equal to 50 and was the mean from at least two independent experiments).

*Apoptosis Assay*

Apoptosis was conducted by flow cytometry using an annexin V-FITC apoptosis detection kit (*BioLegend, USA*) according to the manufacturer’s instructions. Briefly, *AGS* and *KYSE-30* cells (5 × 10^5^) were treated with ALR prepared as concentrations of IC_50_. After 48 h cells were gently trypsinized, washed twice with PBS, and re-suspended in 100 μL of 1X binding buffer. Then, 5 μL of annexin V-FITC and 10 μl of propidium iodide (50 μg/mL) were added. After incubation at room temperature for 15 min in the dark, 400 μL of 1X binding buffer was added to the reaction tube. Annexin V-FITC binding and propidium iodide staining were analyzed by flow cytometer (BD, USA) using the FITC signal detector (FL1) and phycoerythrin emission signal detector (FL3). 

*Real-Time PCR*

The evaluation of mRNA expression level of *TLR4* signaling-related genes including *TLR-4, AKT, ERK1, ERK2*, and *NFκB* in cell lines treated with ALR was performed using quantitative Real-Time PCR (qRT-PCR). After subculture and treatment, total cellular RNA was isolated from the untreated and treated cells using Vizol extraction reagent (Vizpure, Korea) according to the manufacturer’s protocol. Then the quality and quantity of isolated RNA were evaluated using a NANODROP spectrophotometer (Denovix, USA). Subsequently, the RNA was treated with DNase enzyme (Thermo Scientific, USA) and reverse transcribed into cDNA and used as the template for PCR amplification using a reverse transcriptase kit (Thermo Scientific, USA). QRT-PCR was performed by the Applied Biosystems 7300 system (Applied Biosystems, USA). PCR was carried out in a final volume of 20 μL reaction system containing 0.5 μL of each of the forward and reverse primers (Table 2), 10 μL of SYBR green reagent (Vizpure, Korea), 2 μL of cDNA template, and 8.6 μl of nuclease-free water. The PCR cycling was carried out by initial denaturation step at 95 ºC for 3 min followed by 40 cycles at 95 ºC for 10 s, 60 ºC for 15 s, and 72 ºC for 20 s. Relative mRNA expression was measured by the 2^− (ΔCT)^ method, using β-actin as a reference gene.

*Test for cell adhesion*

Cells were re-suspended at 1 ×10^4^ cell/mL in cultural media containing different concentrations of *ALR* (0.03-4 mg/mL) and seeded in 24 wells cultural plates for triplicates. After one hour cells from four wells per concentration with treatment by extracts besides control, wells were collected and the number of unattached cells was determined by counting on a haemocytometer (27, 28).

*Statistical Analysis*

Experimental results are expressed as mean ± SEM. All measurements were replicated three times. The data were analyzed by an analysis of variance (*P < 0.05*). The *IC*_50_ values were calculated in Graph Pad Prism using nonlinear regression analysis.

## Results

*Cytotoxic Activity*

The cytotoxic activity of herbal products was evaluated on 4 different cell lines (*KYSE-30, AGS, HT29* and *L929*) using the MTT assay. The *L929* cells were used as a model for normal cells in this study. Multiple concentrations of herbal extracts were used and *IC*_50_ doses were calculated from the dose–response curve. The results of the cytotoxicity evaluation against esophageal, stomach, colorectal cancer cell lines and normal mouse fibroblast cells are shown in Table 3. According to the results, all treatments displayed a percentage of growth inhibition activity in a dose-dependent manner. Overall, examined herbal extracts had weak cytotoxic activity on HT29 cells, but relatively stronger toxicities against KYSE-30 and particularly on AGS cell lines.The strongest extract for AGS cells was ALR with IC_50_= 10 µg/mL compared to L929 as normal cell line with IC_50 _= 360 µg/mL. For the KYSE-30 cell line, the most cytotoxic extracts were *Arctiumlappa* L. aerial part and root extract with IC_50 _= 100 µg/mL and 200 µg/mL, respectively. Also, *Chrysanthemum morifolium Ramat. *Extract showed IC_50 =_ 500 µg/mL, compared to the L929 normal cells (IC_50_ =1000 µg/mL).

As treatments with ALR caused a remarkable decrease in cell viability of studied cancer cell lines, it was selected for further evaluation of the inflammatory gene expression, apoptotic, and cell adhesion effects just on AGS and KYSE-30 cell lines. 

*Antioxidant assay and total flavonoid/phenolic contents (TPC, TFC, DPPH)*

Total phenolic and flavonoids contents as well as antioxidant activities of *Arctium lappa *aerial parts and root have been shown in Table 4. In the DPPH test, the root total extract exhibited a significant free radical-scavenging activity (IC_50 _= 1250 ‎± ‎0.1 µg/mL) compared to the aerial parts total extract with IC_50 _= 4160‎ ±‎ 0.12 µg/mL (Table 4). 

*Apoptosis Assay*

**Figure 1:**
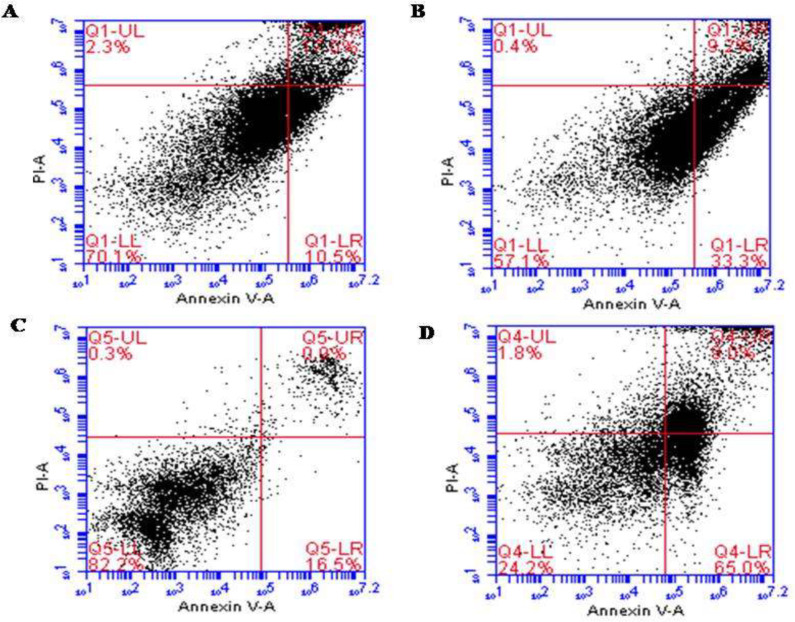
Apoptotic effect of ALR (0.03 mg/mL for 48 h) using Annexin V/PI Flow cytometry analysis of esophageal and stomach cancer cell lines. (A) KYSE-30 cells were double-stained with Annexin V and PI (Control). (B) The rate of apoptosis of KYSE-30 cells treated with AlR. (C) AGS cells were double-stained with Annexin V and PI (Control). (D) The rate of apoptosis of AGS cells treated with AlR

To gain further insights into whether the ALR-mediated reduction in the viability of AGS and KYSE-30 cells related to apoptosis, annexin V and PI staining were conducted. The results confirmed the induction of apoptosis in both cell lines after 48 h treatment of ALR with a concentration of 0.03 mg/mL. Treatment with ALR resulted in the presence of viable (annexin V−/PI−), early apoptotic (annexin V+/PI−), late apoptotic (annexin V+/PI+), and necrotic (annexin V−/PI+) cells. There was a significant difference between treated cells and control for both AGS and KYSE-30 cell lines (*P *< 0.05). In the control, 11.8 ± 1.83% of KYSE-30 cells were apoptotic, whereas 32.1 ± 1.69% of the cells treated with ALR were apoptotic (Figures 1A and 1B). Considering the AGS cells, 15.75 ± 1.06% and 64.65 ± 0.49% of cells were apoptotic in the control and treated cells, respectively (Figures 1C and 1D).

*Real-Time PCR*

**Figure 2:**
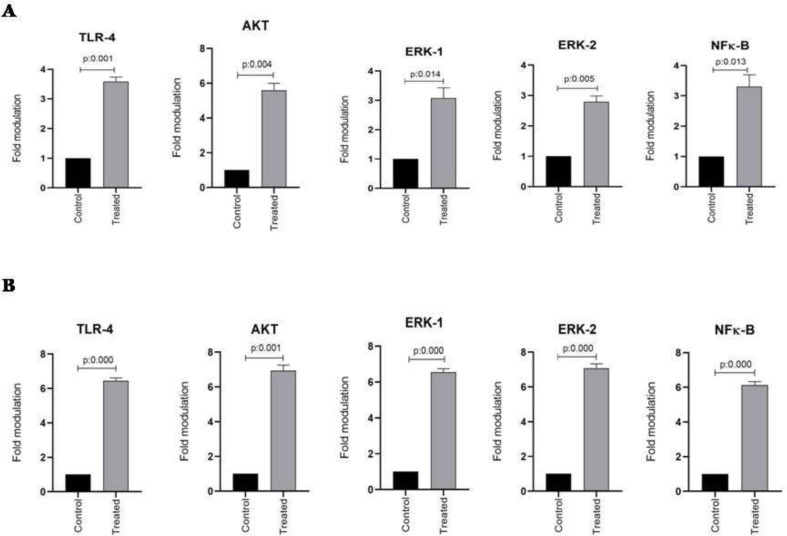
The effect of AlR (0.03 mg/mL) on the expression of TLR-4, Akt, ERK1/2, and NFκB mRNA in esophageal and stomach cell lines. (A) Genes expression in KYSE-30 cell line. (B) Genes expression in AGS cell line. β-actin was used as a loading control. The data were expressed as mean ± SEM. ^*^P < 0.05 *vs*. control group. AlR: Arctiumlappa L. root extract

TLR-4, AKT, ERK1/2, and NFκB mRNA expression in esophageal and stomach cell lines from two studied groups including control and treated cells were evaluated using quantitative real-time PCR assay. The housekeeping gene β-actin was also amplified in all samples and the mRNA expression results were represented as the ratio of TLR-4, AKT, ERK1/2, and NFκB to β-actin. As shown in Figure 2A, the expression of TLR-4, AKT, ERK1/2, and NFκB mRNA significantly increased in the KYSE-30 cell line after treatment with ALR (30 µg/mL) (*P *< 0.05). In addition, this is also true of AGS cell lines, showing the significantly higher mRNA expression levels of TLR-4, AKT, ERK1/2, and NFκB genes (*P *< 0.05) (Figure 2B). It is noteworthy to mention, ALR treatment leads to more up-regulation of mRNA expression of examined genes in AGS in comparison with KYSE-30.To explore any possible correlation between mRNA expressions, all obtained data were analyzed further. Interestingly, significant correlations were observed between TLR-4 and AKT, ERK1/2, and NFκB (*P *< 0.05, r = 1). There was also a correlation between ERK1 and ERK2 mRNA expression (*P *< 0.05, r = 1). Apart from mentioned findings, further investigation was done to determine whether there was any correlation between the percentage of apoptotic cells and the mRNA expression level of TLR4, AKT, ERK1/2 and NFκβ. Of note, a significant correlation was found between percentages of AGS and KYSE-30 apoptotic cells and their related mRNA expression of TLR4, AKT, ERK1/2 and NFκβ (*P *< 0.05, r = 1).

*Cell adhesion*

As cell adhesion is a crucial process in the metastasis of tumor cells, the effect of ALR extract on the adhesion of esophageal and gastric cancer cell lines was investigated in different concentrations including 1000, 2000 and 4000 µg/mL. Interestingly, ALR extract with a concentration lower than 2 mg/mL did not have any effect on the adhesion of KYSE-30 and AGS cells. However, KYSE-30 and AGS cells which are cultivated in the presence of 4000 µg/mL of herbal extract had a diminished capacity of 13% and 5% respectively to attach to a new location, compared to the untreated cells. 

**Table 1:** List of plants screened for cytotoxicity

Plant species	Family	Part used	Collected location	Extract
***Arctiumlappa*** ** L.**	Asteraceae	aerial parts and root	Ziarat	80% ethanol
***Artemisiafragrans *** **Willd.**	Asteraceae	aerial parts	ChaharBagh	“
***Chrysanthemum morifolium *** **Ramat.**	Asteraceae	flowers	Tuskastan	“
***Cichoriumintybus *** **L.** ***Cuscutaepithymum *** **L** ***.***	Asteraceae Convolvulaceae	aerial parts and root aerial parts	ChaharBagh -	“ “
***Equisetum arvensis *** **L.**	Equisetaceae	leaves	Tuskastan	“
***Myrtuscommunis *** **L.**	‎Myrtaceae	aerial parts	MaravehTappeh	“
***Urticadioica*** ** L.**	Urticaceae	Leaves and root	Ziarat	“
***Artemisia annua***	Asteraceae	leaves	Ziarat	“

**Table 2:** Sequences of primers used for real time PCR

Gene	Forward primer	Reverse primer	Amplicon size (bp)
**NF-κB P65**	ATCCCATCTTTGACAATCGTGC	CTGGTCCCGTGAAATACACCTC	153
**AKT2**	TGAAAACCTTCTGTGGGACC	TGGTCCTGGTTGTAGAAGGG	145
**ERK1**	ACCCTGGAAGCCATGAGAGA	GGCGGAGTGGATGTACTTGA	150
**ERK2**	TTCCAACCTGCTGCTCAACA	TCTGTCAGGAACCCTGTGTGAT	102
**TLR4**	TGGAAGTTGAACGAATGGAATGTG	ACCAGAACTGCTACAACAGATACT	148
**β-actin**	CCTTCCTGGGCATGGAGTCCT	TGGGTGCCAGGGCAGTGAT	174

**Table 3:** MTT Assay of Extracts against L-929, AGS, HT-29, and KYSE-30 Cell Lines during 48. h

Extracts IC_50 _(µg/mL)				
	L-929	AGS	HT-29	KYSE-30
***Arctiumlappa*** ** L. root **	360	10	2030	200
***Arctiumlappa*** ** L. aerial part **	200	100	2100	100
***Artemisiafragrans *** **Willd. **	100	2100	2600	1200
***Chrysanthemummorifolium *** **Ramat.**	1000	190	500	500
***Cichoriumintybus *** **L. root**	100	-	600	200
***Cichoriumintybus *** **L. aerial part**	1600	300	1100	1020
***Cuscutaepithymum *** **L**	3300	-	700	300
***Equisetum arvensis *** **L.**	10000	1300	700	600
***Myrtuscommunis *** **L. **	3110	2530	5500	5100
***Urticadioica*** ** L. root **	2500	6300	1300	-
***Urtica dioica*** ** L. aerial part **	1200	3250	1100	-
***Artemisiannua***	500	2400	1500	-

Each IC_50_ are reported as mean IC50 values ± SEM from 3 independent experiments.

**Table 4:** Antioxidant activity, total flavonoid and phenolic contents of *Arctiumlappa *L

Samples	DPPHIC_50_ (mg/mL)	Total flavonoids (mg QUE/g ofsample)	Total phenolics (mg GAE/g ofsample)
**Aerial parts**	4.16‎ ± ‎0.12	48.06 ± 1.4	116.4 ± 1.1
**Root**	1.25 ‎± ‎0.10	69.03 ± 0.7	215.8 ± 0.3

## Discussion

Gastrointestinal cancers as one of the public health concerns are the most common cancers in some areas of the world (1). Since cancer therapies with chemical drugs are accompanied by resistance and side effects, many studies have focused on using herbal medicines due to the multi-beneficial effects of natural products in comparison with conventional treatments. The main objective of this study was to evaluate the cytotoxic effects of some herbal extracts on AGS, KYSE-30, HT29 cancer cell lines, and L-929 as a control normal cell. In this study, the MTT assay represented that *Cichoriumintybus*, *Urticadioica*, and especially *Arctium lappa* had the strongest cytotoxicity effects. The total root extract of *Arctium lappa* at 10 and 200 µg/mL resulted in a 50% decrease (IC_50_) in the proliferation of AGS and KYSE-30 cell lines, respectively. 

Nevertheless, the value of IC_50_ for the aerial part of *Arctium lappa* was 100 µg/mL for AGS and 100 µg/mL for the KYSE-30 cell line. The IC_50_ value for both aerial and root extract of *A. lappa *was evaluated to be about 2000 µg/mL for the HT29 cell line. Our results showed that the extract of root and aerial parts of *A. lappa *revealed a significant cytotoxic effect on each cell line. Additionally, the ALR was the most potent extract on AGS cell lines, while its aerial part extract was more effective on KYSE-30. Both aerial parts and root extracts of *A. lappa*’seffect on HT29 were less than the two other cell lines. These findings are in line with previous studies that reported cytotoxic activity of herbal extracts in different cancer cell lines with different IC_50 _in each cell line (29-32). In addition, L-929 was considered in the study as a control group and IC_50_ values of L-929 were remarkably more than cancer cell lines. 

In our study, HT29 cells were relatively less sensitive to studied herbal concentrations compared to the other cancer cells. As many previous studies have mentioned, the major obstacle of colorectal cancer is the appearance of resistance to different chemical compounds (1, 2). Overall, obstacles and difficulties in treating cancer, particularly colon cancer, underscores the need to develop novel therapeutic agents or adjuvant to complete the effects of chemical drugs (33-35). Consistent with this evidence, the crude extract of *Rosmarinusofficinalis *L. fruit displayed a cytotoxic effect due to its apoptosis properties on AGS and KYSE-30 gastrointestinal cell lines. It was also observed that gastric cancer cells (AGS) were more sensitive to the cytotoxic effect of the extract (36).

In the next part of the study, ALR was selected for further investigations on the AGS and KYSE-30 cell lines, based on its efficacy and cytotoxic effect. This study demonstrated that ALR revealed high antioxidant activity, flavonoid and phenolic contents. Also, the apoptosis effect of ALR in both AGS (6-fold compared to control) and KYSE-30 cell lines (3-fold compared to control) within 48 h. Gastric cancer cells (AGS) exhibited higher sensitivity in agreement with the results of the cytotoxicity test. To determine whether ALR treatment can affect the expression of TLR-4 and related genes in AGS and KYSE-30 cells, Real-time PCR was performed to determine gene expression at the mRNA level. The results showed that mRNA expression of target genes was upregulatedfollowing treatment with ALR for 48 h in AGS and KYSE-30 cell lines. This finding is in accordance with a study that demonstrated that Arctigenin (ARG), one of the most active ingredients attributed to ALR could increase the mRNA expression of TLRs including TLR4 (37).

Interestingly, ALR significantly increased the expression of TLR-4 and the mRNA expression level of other genes as well as the apoptotic effect in AGS more often in comparison with the KYSE-30 cell line. Hence, these may also show the potent effect of mentioned genes to induce apoptosis and cytotoxicity in AGS cell lines. This beneficial feature of *Arctium lappa* is very important because it implies that treatment with *Arctium lappa* can enhance the apoptosis of gastric and esophageal cancerous cells through up-regulation of TLR-4 and NFκB. In our previous study on colon cancer cells, we demonstrated that LPS had a synergistic effect with 5-FU on up-regulation of TLR-4 expression on the surface of HCT116 cells (colon cancer cells). Also, LPS enhanced 5-FU-induced apoptosis in colon and breast cancer cells (38, 39). These results were in line with Mikami *et al.*’s study which illustrated that TLR-4 inhibits tumor growth in cutaneous squamous cell carcinoma (SCC), and TLR-4 knockdown enhances the migration and invasion in SCC cells (40). On the other hand, recent studies have demonstrated that the TLR-4 antagonist- resatorvid- blocks solar UV-induced skin tumorigenesis in mice *ex-vivo* and *in-vivo* (41). These reports have indicated that TLR4 may be a suitable target to prevent photo-carcinogenesis. In contrast with our previous study, Zhang *et al.* in a study on prostate cancer cells showed that ligation of TLR-4 with lipopolysaccharide (LPS) abrogated docetaxel-induced growth suppression in PC-3 prostate cancer cells (42). 

Rebeca Santaolalla in a study on the breast cancer cells also showed that TLR-4 enhances migration of breast cancer cells, via PI3K/AKT/GSK3β, and promotes transcription of downstream-catenin target genes (MMP7, MMP9, and VEGFA), leading to breast cancer metastasis. The results suggest that the AKT/GSK3β signaling pathway may indicate a viable clinical treatment target in breast cancer (43).

Recent studies have shown that the PI3K/AKT pathway modulates cell survival, cell cycle progression and cellular growth, and hyperactivation of this pathway in various cancers increases proliferation and reduces apoptosis. MAPKs, a family of serine/threonine kinases, including p38 MAPK, JNK, and ERK also play important roles in apoptosis and cell proliferation in a variety of cancers, Therefore, they may be considered as possible therapeutic targets (44). Our results showed that treatment with the extract significantly increased apoptosis and upregulated the expression of TLR-4, AKT and ERK in AGS and KYSE-30 cells.

Overall, TLR-4 may promote or inhibit tumor, depending on the tumor type, the stimulator of the receptor, and also the situation of the host. Subsequently, the molecules in downstream signaling of TLR-4 like AKT and ERK are also involved in the anti-tumor or tumorigenesis effects of TLR-4. In the current study treatment with ALR extract enhanced cytotoxicity and apoptosis in AGS and KYSE-30 cell lines. Up-regulation of the TLR-4, AKT, and ERK was also shown in AGS and KYSE-30 cell lines when treated with ALR extract.

In the subsequent experiments, a cell adhesion test was performed to determine the effect of ALR on the adhesion properties of AGS and KYSE-30 cell lines. The ability of cells to adhere is essential for the execution of their specific functions. On the other hand, it is important to the assessment of the invasiveness of cancer cells. The results showed that ALR has no effect on the adhesion of these cells at low concentrations including 1 and 2 mg/ml. However, at a concentration of 4 mg/ml, ALR treatment reduced the capacity of cell adhesion of AGS and KYSE-30 cell lines. In line with our study, Lee et al. showed that treatment with *Arctium lappa* suppressed up-regulation of intercellular adhesion molecule (ICAM)-1, vascular cell adhesion molecule (VCAM)-1, and E-selectin in the aorta of rats. These results suggested the anti-inflammatory effect of the extract in the rat model (45).

## Conclusion

*Arctium lappa* L. root extract with cytotoxicity effect on cancer cell lines enhances apoptosis and up-regulates the expression of TLR-4 and AKT/ERK in the downstream pathway of the TLR-4 cell signaling and decreases tumor cells adhesion. These properties may suggest the ALR extract as an alternative or complement in cancer therapy even though intense photochemistry and *in vivo *studies still need to be done on this plant.
